# Reduced Calcium Signaling Is Associated With Severe Graft-Versus-Host Disease: Results From Preclinical Models and From a Prospective EBMT Study

**DOI:** 10.3389/fimmu.2020.01983

**Published:** 2020-08-11

**Authors:** Katarina Riesner, Steffen Cordes, Christophe Peczynski, Martina Kalupa, Constanze Schwarz, Yu Shi, Sarah Mertlitz, Jörg Mengwasser, Steffie van der Werf, Zinaida Peric, Christian Koenecke, Helene Schoemans, Rafael F. Duarte, Grzegorz W. Basak, Olaf Penack

**Affiliations:** ^1^Department of Hematology, Oncology, and Tumor Immunology, Charité Universitätsmedizin Berlin, Berlin, Germany; ^2^EBMT Transplant Complications Working Party, Paris, France; ^3^EBMT Statistical Unit, Paris, France; ^4^Department of Surgery, Charité Universitätsmedizin Berlin, Campus Charité Mitte/Campus Virchow Clinic, Berlin, Germany; ^5^EBMT Data Office, Leiden, Netherlands; ^6^University Hospital Center Rebro, Zagreb, Croatia; ^7^Hannover Medical School, Hanover, Germany; ^8^UZ Leuven, Leuven, Belgium; ^9^Hospital Universitario Puerta de Hierro, Madrid, Spain; ^10^Medical University of Warsaw, Warsaw, Poland; ^11^Berlin Institute of Health, Berlin, Germany

**Keywords:** graft-versus-host-disease, stem cell transplantation, calcium, GPRC6a, GVHD mouse models, EBMT study

## Abstract

Despite its involvement in various immune functions, including the allogeneic activation of T-lymphocytes, the relevance of calcium (Ca^2+^) for GVHD pathobiology is largely unknown. To elucidate a potential association between Ca^2+^and GVHD, we analyzed Ca^2+^-sensing G-protein coupled receptor 6a (GPRC6a) signaling in preclinical GVHD models and conducted a prospective EBMT study on Ca^2+^ serum levels prior alloSCT including 363 matched sibling allogeneic peripheral blood stem cell transplantations (alloSCTs). In experimental models, we found decreased *Gprc6a* expression during intestinal GVHD. GPRC6a deficient alloSCT recipients had higher clinical and histopathological GVHD scores leading to increased mortality. As possible underlying mechanism, we found increased antigen presentation potential in GPRC6a^–/–^ alloSCT recipients demonstrated by higher proliferation rates of T-lymphocytes. In patients with low Ca^2+^ serum levels (≤median 2.2 mmol/l) before alloSCT, we found a higher incidence of acute GVHD grades II-IV (HR = 2.3 Cl = 1.45–3.85 *p* = 0.0006), severe acute GVHD grades III-IV (HR = 3.3 CI = 1.59–7.14, *p* = 0.002) and extensive chronic GVHD (HR = 2.0 Cl = 1.04–3.85 *p* = 0.04). In conclusion, experimental and clinical data suggest an association of reduced Ca^2+^ signaling with increased severity of GVHD. Future areas of interest include the in depth analysis of involved molecular pathways and the investigation of Ca^2+^ signaling as a therapeutic target during GVHD.

## Introduction

Calcium (Ca^2+^) signaling is involved in the regulation of various immune functions, including B- and T-cell activation, differentiation, T-lymphocyte-mediated cytotoxicity and cytokine gene expression (1). It has been shown to modulate the allogeneic activation of T-lymphocytes by antigen presenting cells (2, 3) implicating a role for graft-versus-host disease (GVHD) pathogenesis. Additionally, Ca^2+^ is known as “danger-associated molecular pattern” (DAMP) and plays an important role in the activation of T-cells, dendritic cells and the multi-protein complex of the NOD-like receptor protein (NLRP3) inflammasome (4). During conditioning prior to allogeneic stem cell transplantation (alloSCT), with radiation and/or chemotherapy, and later during GVHD increased cell death of immune and tissue cells occurs. This increased cell death can lead, among other things, to enhanced Ca^2+^ levels. A locally increased extracellular Ca^2+^ concentration can induce the activation of the NLRP3 inflammasome via the calcium sensing receptor and the G-protein coupled receptor 6a (GPRC6a) (4). Several studies revealed a significant role of DAMPs and the NLRP3 inflammasome in GVHD morbidity and mortality (5–8). While NLRP3 function has been studied in GVHD, upstream regulatory functions of GPRC6a in GVHD remain to be elucidated. GPRC6a is a class C, group 6, subtype A G-protein coupled receptor which can be directly activated or positively modulated by Ca^2+^ in concentrations above 5 mM; and was shown to be involved in the regulation of inflammation, metabolism and endocrine functions (9). In addition, GPRC6a was identified in a genome-wide association study as a novel loci associated with C-reactive protein levels, a general biomarker for systemic inflammation (10).

The role of Ca^2+^ during GVHD is mostly unknown. Thangavelu et al. showed an effect of genetically and inhibitor-induced diminished intracellular Ca^2+^ levels on donor T-cell survival during GVHD in preclinical models (11). Based on prior knowledge and the fact that Ca^2+^ is a routine laboratory parameter in patients undergoing alloSCT due to frequently arising disturbances in calcium metabolism in these patients, there is a strong rationale to study Ca^2+^ and its association to GVHD. We performed experiments in preclinical GVHD models using GPRC6A deficient mice; and the Transplant Complications Working Party (TCWP) of the European Society for Blood and Marrow Transplantation (EBMT) performed a prospective, multicenter and non-interventional clinical study following the hypothesis that Ca^2+^ is correlated to the occurrence of GVHD.

## Materials and Methods

### Preclinical Data

#### Study Design and Statistics

Sample size for GVHD experiments was calculated by estimation of time point–specific analyses based on the Student’s *t*-test assuming 80% power and 0.05 2-sided level of significance. Experiments with at least 5 animals per group (wildtype transplanted [WT] and GPRC6a deficient transplanted [GPRC6a**^–^**^/^**^–^**]) were performed 2 times. Animals were randomized, including equal distribution of weight status and mixed housing of different transplanted animals. For subsequent analyses, transplant conditions were encoded. All experiments were approved by the Regional Ethics Committee for Animal Research (State Office of Health and Social Affairs, Berlin).

Survival data of at least 10 animals per group (WT and GPRC6a**^–^**^/^**^–^** transplanted) were performed 2 times and analyzed using the Kaplan-Meier method and compared with the Mantel-Cox log-rank test. For all other data, the Student’s unpaired *t*-test (two-tailed) was used if not indicated differently. Normality tests and F test confirmed Gaussian distribution and equality of variance between different groups. Values are presented as mean ± standard deviation (SD). Values of *P* < 0.05 were considered statistically significant. All statistical analyses were performed using GraphPad Prism software (GraphPad Software Inc, La Jolla, CA, United States).

#### Mice

Female C57BL/6 (B6) (H2^*b*^) and BALB/c (H2^*d*^) mice (10–12 weeks old) were purchased from Charles River Laboratories (Sulzfeld, Germany) and from Janvier (St. Berthevin Cedex, France), respectively. Female GPRC6a deficient (GPRC6a^–/–^) mice on B6 background were generated by Hans Bräuner-Osborne, provided by Ulf Wagner (University Hospital Leipzig, Germany) and were bred by and obtained from the central animal unit of the Charité University Medicine. Mice had access to food and water *ad libitum*.

#### GVHD Experiments

Our alloSCT acute GVHD models are characterized well (12–16). We performed alloSCTs in the BALB/c→B6 model using GPRC6a^–/–^ mice as donors and recipients. GPRC6a^–/–^ or B6→BALB/c: Recipient mice received 800 cGy total body irradiation from a 137Cs source as a split dose with a 4-h interval and were injected intravenously (i.v.) with 5 × 10^6^ bone marrow (BM) cells and 0.5 × 10^6^ splenic T-cells. BALB/c→ GPRC6a^–/–^ or B6: Recipient mice received 1200 cGy total body irradiation from a 137Cs source as a split dose with a 4-h interval and were injected i.v. with 0.75 × 10^7^ BM cells and 1 × 10^6^ splenic T-cells. BM was flushed from the tibia and femur, and single-cell suspension was prepared in phosphate-buffered saline (PBS)/2% fetal calf serum/1 mM EDTA by gently passing through a 23-G needle and over a 70 μm cell strainer (BD Biosciences, San Jose, CA, United States). Splenic T-cell suspension was obtained using the Pan T-cell Isolation Kit II (Miltenyi Biotec, Bergisch Gladbach, Germany). T-cell purity was analyzed by CD3 staining and FACS analysis. For *Gpcr6a* expression analysis, we used the established chemotherapy-based minor mismatch model LP/J→B6 as described in (16). Mice were individually scored twice a week for 5 clinical parameters (posture, activity, fur, skin, and weight loss) on a scale from 0 to 2. Clinical GVHD score was assessed by summation of these parameters. Survival was monitored daily.

#### Quantitative Real-Time PCR

A total of 2 μg RNA from colon of syngeneic and allogeneic transplanted mice at day + 15 after alloSCT and complementary DNA, obtained using the RNeasy Mini Kit (Qiagen, Venlo, Netherlands) and the QuantiTect Reverse Transcription Kit (Qiagen) following the manufacturer’s instructions, were amplified (50°C, 2 min; 95°C, 10 min; 49 cycles of 95°C, 10 s; 60°C, 1 min) on DNA Engine Opticon (Bio-Rad, Hercules, CA, United States) using the TaqMan Gene Expression Master Mix (Life Technologies) and GPRC6a primers and probe from BioTez GmbH (Berlin, Germany). Data were analyzed with Opticon Monitor 3.1 analysis software (Bio-Rad) and the comparative CT Method (ΔΔCT Method).

#### Flow Cytometry

Single-cell suspensions of blood, BM, lymph nodes, spleen and thymus were prepared. Lymph nodes, splenocytes and thymocytes were passed through a 40-μm cell strainer. Erythrocytes were lysed with ammonium chloride. Cells were washed twice and stained for 20 min at 4°C in PBS/0.5 mM EDTA/0.5% BSA with the following rat mAbs from BD Biosciences: anti-H2kd (SF1-1.1-FITC and PE), anti-CD8a (53-6.7-APC), anti-Ly-6G and Ly6C/Gr1 (RB6-8C5-APC), anti-CD45R/B220 (RA3-6B2-PerCP-Cy5.5), anti-CD25 (PC61-PerCP-Cy5.5), anti-CD4 (RM4-5-PE-Cy7), anti-CD80 (16-10A1-PE), anti-CD86 (GL1-PerCP-Cy5.5), anti-CD11c (HL3-PE-Cy7), anti-CD3e (145-2C11-APC-Cy7) and anti-CD11b (M1/70-APC-Cy7). For staining of regulatory T-cells, Anti-Mouse/Rat FoxP3 Staining Set APC (eBioscience, San Diego, CA, United States) was used following the manufacturer’s instructions. Samples were analyzed by BD FACS Canto II (BD Biosciences) and FlowJo 7.6.5 Software (TreeStar Inc., Ashland, OR, United States).

#### Mixed Leukocyte Reaction

Dendritic cells were isolated from spleen of GPRC6a^–/–^ and B6 mice using a CD11c + isolation kit and splenic T-cells from BALB/c mice were obtained using the mouse Pan T-cell isolation Kit II (Miltenyi Biotec) according to the manufacturer’s instructions. T-cells were loaded with carboxyfluorescein diacetate succinimidylester (CFSE, Thermo Fisher Scientific, Waltham, MA). 2.5 × 10^4^ dendritic cells (activators) and 2.5 × 10^5^ T-cell (responders) were incubated for 96 h at 37°C and 5% CO_2_. Total cell counts being negative for CFSE and positive for H2kd were analyzed via flow cytometry. Proliferating cells were determined as cells, showing no CFSE load compared to control samples without T-cell stimulation at time point 0 h.

#### *In vivo* T-Cell Proliferation Assay

GPRC6a-/- and B6 recipients received 1200 cGy total body irradiation from a 137Cs source. CD3 + lymphocytes were isolated from spleens of BALB/c donors and enriched by Pan T-Cell isolation kit (Milteny Biotec). 4 × 10^6^ CFSE-loaded CD3 + cells were injected i.v. into the tail vein. 72 h later, mice were sacrificed and T-cells from spleen, thymus, blood and lymph nodes were isolated and analyzed for proliferation by flow cytometry. Total cell counts being negative for CFSE and positive for H2kd were analyzed via flow cytometry. Proliferating cells were determined as cells, showing no CFSE load compared to control samples without T-cell stimulation at time point 0 h.

### Clinical Data

#### Data Source, Study Design, and Data Collection

We asked EBMT centers performing more than 50 alloSCT per year if they were willing to participate in this prospective study. 17 centers in ten countries agreed to participate. Data collection for the EBMT registry was approved by the IRB and/or Ethics Committee in all centers. Data were prospectively collected between 6/2014 and 3/2018. Consecutive alloSCT recipients with acute leukemia, lymphoma or myelodysplastic syndrome (MDS) receiving a first matched sibling alloSCT from peripheral blood, regardless of conditioning, were eligible, provided they had signed an informed consent document that permitted sharing of clinical data according to national rules. Basic data on patient and disease characteristics as well as longer term follow up was taken from minimal essential data (MED-A) forms, which are submitted from all consecutive patients to the central EBMT registry. In addition, we designed registration and MED-B/C forms that were prospectively collected and specific to this study. The MED-B/C form contained detailed information on calcium serum levels prior to alloSCT, patient characteristics, infectious- as well as non-infectious complications, GVHD staging, morbidity and mortality. Total Calcium (including bound Ca^2+^ to proteins, mainly albumin, and anions; and free ionized Ca^2+^) serum levels were determined photometrically and corrected for albumin levels according to Payne’s formula [Ca _*corr*_ [mmol/L] = Ca _*measured*_ [mmol/L] – [0.025 × Albumin [g/L] + 1] at time of hospital admission for alloSCT directly before start of conditioning therapy. Treatment teams completed the specific forms at the time of registration and at day + 100 after alloSCT.

#### Endpoints and Statistical Analyses

Patient, disease, and transplant-related characteristics for the two cohorts [calcium serum levels prior to alloSCT above median (>2.2 mmol/l)/calcium levels below median (<2.2 mmol/l)] were compared by using χ2 statistics for categorical variables and the Mann-Whitney test for continuous variables. Primary endpoint was the incidence of acute GVHD. Secondary endpoints were relapse incidence (RI), non-relapse mortality (NRM), overall survival (OS), progression free survival (PFS) and the incidence of chronic GVHD. PFS was defined as survival with no evidence of relapse or progression. RI was defined as the probability of having had a relapse during follow up time. Death without experiencing a relapse was a competing event. NRM was defined as death without evidence of relapse or progression. OS was defined as the time from alloSCT to death, regardless of the cause. Acute GVHD was graded according to the modified Seattle-Glucksberg criteria (17) and chronic GVHD according to the revised Seattle criteria (18). Cumulative incidence was used to estimate the endpoints of NRM, RI, acute, and chronic GVHD to accommodate for competing risks. To study acute and chronic GVHD, we considered relapse and death to be competing events. Probabilities of OS and PFS were calculated using the Kaplan–Meier method. Univariate analyses were done using the Gray test for cumulative incidence functions and the log rank test for OS and PFS. A Cox proportional hazards model was used for multivariate regression. All variables differing significantly between the 2 groups or factors associated with one outcome in univariate analysis were included in the Cox model. The following variables entered the multivariate models as possible confounders: age, sex mismatch between recipient and donor, diagnosis, disease status, Karnofsky score, number of CD34 cells given, intensity of conditioning (EBMT definition: myeloablative conditioning (MAC) was defined as TBI > 6 Gray or oral busulfan > 8 mg/kg or intravenous busulfan > 6.4 mg/kg), type of GVHD prophylaxis, ATG use, time from diagnosis to transplant, year of transplant and CMV status. As the number of variables was too high regarding the number of events, a stepwise selection using Akaike information criterion (AIC) was run for all the confounding factors. The difference between the two cohorts was then assessed in the final selected model.

Results were expressed as the hazard ratio (HR) with the 95% confidence interval (95% CI). Proportional hazards assumptions were checked systematically for all proposed models using the Grambsch-Therneau residual-based test. All tests were 2-sided. The type I error rate was fixed at 0.05 for the determination of factors associated with time-to-event outcomes. Statistical analyses were performed in November 2018 with R 3.4.2 (R Core Team (2017). R: A language and environment for statistical computing. R Foundation for Statistical Computing, Vienna, Austria^1^.

## Results

### GPRC6a in Preclinical GVHD

**FIGURE 1:**
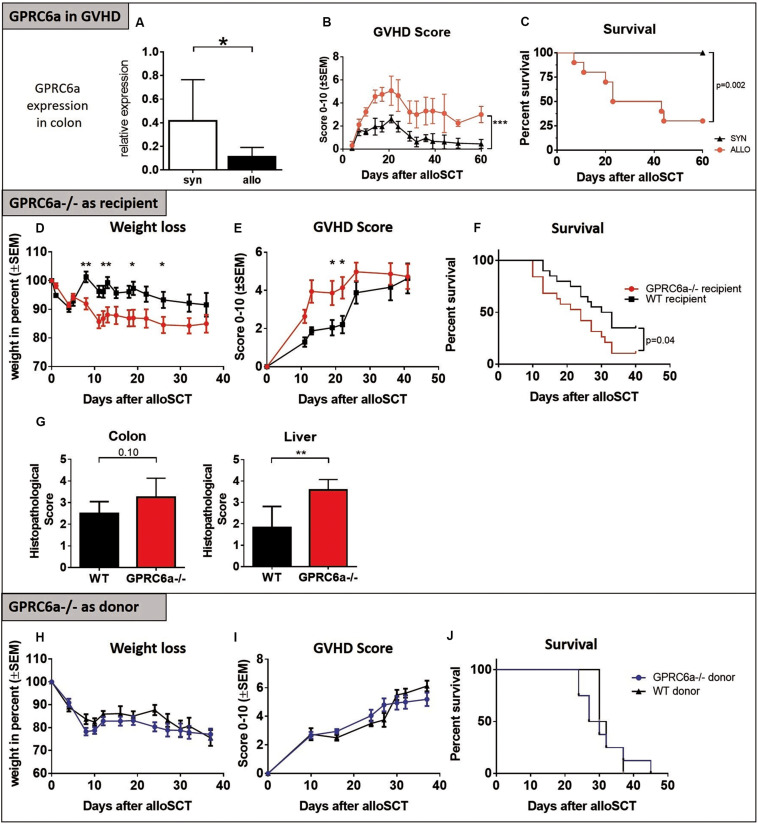
GPRC6a in preclinical GVHD. **(A)** Reduced *Gprc6a* expression in colon of allogeneic (allo) compared to syngeneic (syn) transplanted mice during acute GVHD at day + 15 after LP/J→B6 SCT (*n* = 10 per group). Gene expression level was normalized to *Gapdh* expression and is shown relative to gene level of a reference naïve control. **(B,C)** GVHD Scores and survival in LP/J→B6 alloSCT. **(D–G)** *GPRC6a deficient* (^–/–^*) mice as alloSCT recipients*: BALB/c→GPRC6a^–/–^ alloSCT. GPRC6a^–/–^ mice showed more GVHD-dependent weight loss **(D)**, higher GVHD-Scores **(E)**, decreased survival **(F)** (*n* = 20 per group) and higher histopathological GVHD Scoring determined on HE sections of colon and liver at day + 15 after alloSCT **(G)** (*n* = 6 per group). B6 wildtype (WT) recipient mice served as control. **(H–J)** *GPRC6a*^–/–^ *mice as alloSCT donors:* GPRC6a^–/–^→BALB/c alloSCT. BALB/c recipients (*n* = 10) transplanted from GPRC6a^–/–^ donor mice showed no differences in weight loss **(H)**, GVHD Score **(I)** and survival **(J)** compared to BALB/c recipients (*n* = 5) transplanted from B6 WT donor mice. In panels F and J, *P* values were calculated by using the log-rank test. Error bars indicate mean ± SD. *P < 0.05; **P < 0.01; by Student’s *t*-test (two-tailed). Mice experiments were performed twice.

In experimental acute GVHD, we found significantly decreased *Gprc6a* expression levels in the colon of allogeneic transplanted recipients at day + 15 after alloSCT (Figure 1A) during established acute GVHD (Figures 1B,C); feasibly suggesting defects in calcium induced signaling during acute GVHD. No expression in skin and liver was detectable with the available detection methods. Generally, diverse studies have shown that GPRC6a is widely expressed albeit at relatively low levels making detection challenging (9). To further analyze the effect of GPRC6a on GVHD, we performed alloSCTs in the BALB/c→B6 murine GVHD model using mice deficient for GPRC6a (GPRC6a^–/–^, B6 background) either as recipients or donors of alloSCT. In line with the expression data, GPRC6a^–/–^ alloSCT recipients exhibited higher GVHD morbidity and mortality. GPRC6a^–/–^ alloSCT recipients had significantly increased weight loss (Figure 1D), GVHD scores (Figure 1E) and mortality (Figure 1F). GVHD typical target organ damage was also more pronounced in GPRC6a^–/–^ alloSCT recipients, exhibiting higher histopathological scores, significantly in liver and non-significantly in colon, compared to wildtype (WT) alloSCT recipients at day + 15 after alloSCT (Figure 1G). The use of GPRC6a^–/–^ as alloSCT donors did not result in changed GVHD morbidity and mortality compared to WT donors. Weight loss, GVHD scores and survival were similar in both groups (Figures 1H–J). We conclude that GPRC6a signaling in the alloSCT recipient but not in the alloSCT donor may play a role during acute GVHD.

### Inflammatory Cells in GPRC6a^–/–^ alloSCT Recipients

**FIGURE 2:**
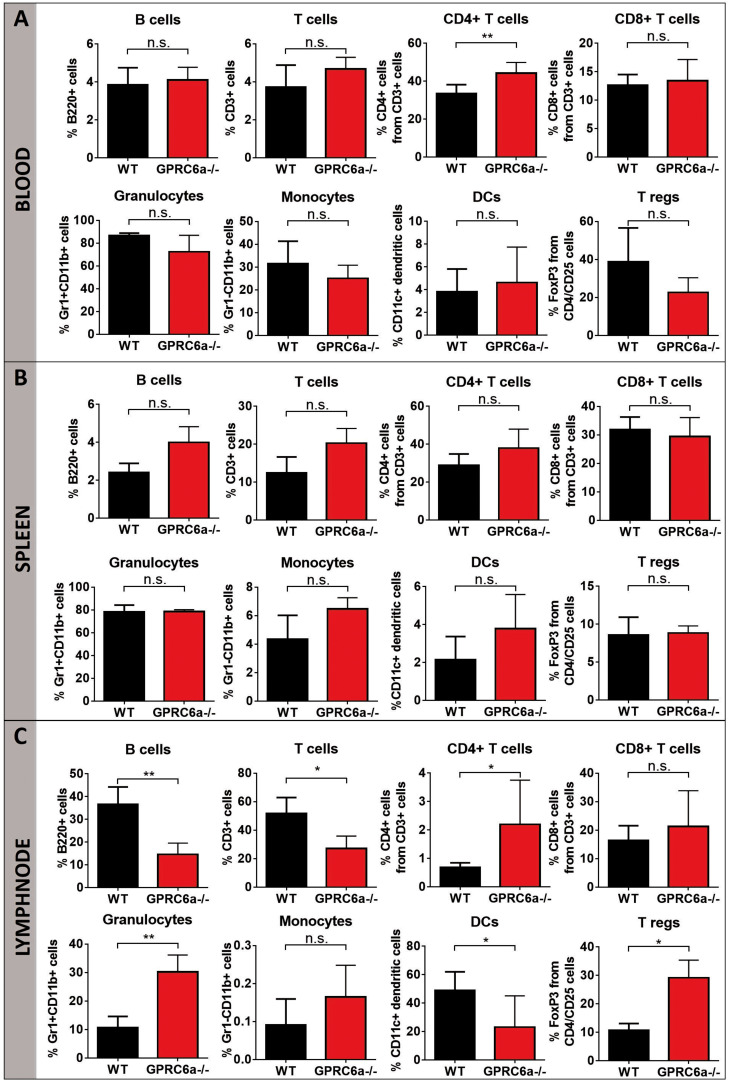
Inflammatory cells in GPRC6a^–/–^ recipient alloSCT mice during GVHD. FACS analysis of CD3, CD4, CD8, B220, CD11b, CD11c, Gr1, CD25, FoxP3 was performed in peripheral blood **(A)**, spleen **(B)** and axillary lymph nodes **(C)**. Samples from BALB/c→GPRC6a^–/–^ at day + 15 after alloSCT (*n* = 6). Wildtype (WT) B6 recipient mice served as control (*n* = 5). Mice experiments were performed twice. Error bars indicate mean ± SD. *P < 0.05; **P < 0.01; n.s., not significant by Student’s *t*-test (two-tailed).

**FIGURE 3:**
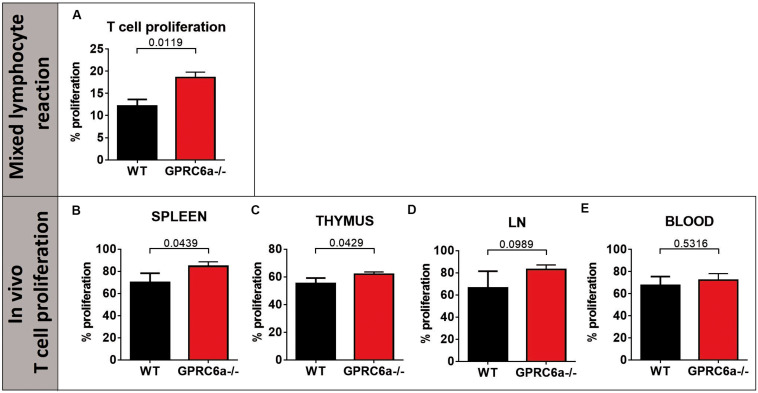
Proliferation of allogeneic T-cells by GPRC6a^–/–^ cells. **(A)** *T-cell proliferation in vitro (Mixed lymphocyte reaction):* Percentage of proliferated T-cells after 96 h incubation with dendritic cells analyzed via CFSE in flow cytometry. T-cells were isolated from BALB/c mice, dendritic cells from B6 wildtype (WT) and GPRC6a^–/–^ mice (*n* = 3 per group). **(B–E)** *T-cell proliferation in vivo:* 4 × 10^6^ CFSE labeled T-cells from BALB/c mice (H2kd +) were transplanted in irradiated B6 WT and GPRC6a^–/–^ mice (*n* = 4 per group). After 72 h, donor (H2kd +) T-cell proliferation was assessed via CFSE in flow cytometry in spleen **(B)**, thymus **(C)**, axillary lymph nodes **(D)**, and peripheral blood **(E)**. Proliferating cells were determined as cells, showing no CFSE load compared to control samples without T-cell stimulation at time point 0 h. Error bars indicate mean ± SD. *P* < 0.05; significant by Student’s *t*-test (two-tailed).

To elucidate the higher acute GVHD morbidity and mortality in GPRC6a^–/–^ alloSCT recipients, we analyzed peripheral blood, spleen and axillary lymph nodes for inflammatory cells during pronounced GVHD symptoms at day + 15 after alloSCT. In GPRC6a^–/–^ alloSCT recipients, we found significantly increased CD4^+^ T-cells in blood and lymph nodes (Figures 2A,C). In spleen, no significant changes compared to WT B6 alloSCT recipients were detected (Figure 2B), however, in lymph nodes, GPRC6a^–/–^ alloSCT recipients exhibited significantly decreased B-cell and dendritic cells and significantly increased granulocytes and regulatory T-cells (Figure 2C). As underlying mechanism, we found increased antigen presentation potential in GPRC6a^–/–^ alloSCT recipients demonstrated by higher proliferation rates of T-lymphocytes. In mixed lymphocyte reaction, BALB/c T-cells showed higher proliferation after 96 h incubation with GPRC6a^–/–^ dendritic cells as compared to WT dendritic cells (Figure 3A). To analyze the proliferation potential of allogeneic T-cells *in vivo*, we transplanted CFSE-labeled BALB/c (H2k^*d*^) T-cells in either irradiated WT B6 or GPRC6a^–/–^ mice (H2k^*b*^). After 72 h, allogeneic T-cells in GPRC6a^–/–^ mice showed a significantly increased proliferation compared to WT B6 mice in spleen and thymus (Figures 3B,C), a non-significant increase in axillary lymph nodes (Figure 3D) and no significant changes in the peripheral blood (Figure 3E).

We conclude that the potential to induce allogeneic T-cell proliferation is increased in GPRC6a^–/–^ antigen presenting cells as compared to WT antigen presenting cells, providing a possible mechanism for the observed increased acute GVHD in GPRC6a^–/–^ alloSCT recipients.

To further elucidate the clinical significance of these preclinical findings showing a possible correlation between Ca^2+^ and the occurrence of GVHD, we analyzed patient data from a prospective, multicenter and non-interventional clinical study performed by the Transplant Complications Working Party of the EBMT.

### Patients, Transplant Characteristics, and Serum Calcium Measurement

The entry criteria for analysis of primary and secondary endpoints were fulfilled in 363 patients. We used the last total Ca^2+^ serum level that was measured in the individual patients before start of conditioning. The Ca^2+^ cut off point was determined at 2.2 mmol/l (median of measured total Ca^2+^ levels), resulting in a high (>median 2.2mmol/l) and a low (≤median 2.2mmol/l) Ca^2+^ serum level group. Total Ca^2+^ serum levels were corrected for albumin levels; of note we found in our population a strong correlation between calcium and albumin values (*r* = 0.56, *p* < 0.0001). The main patients and transplant characteristics that were included in the analysis of overall survival are described in Supplementary Table 1. The majority of parameters were balanced between the two cohorts. However, a higher percentage of patients in complete remission (CR) was observed in the group of patients with Ca^2+^ above median before alloSCT. The frequency of patients with reduced-intensity conditioning (RIC) versus myeloablative conditioning differed significantly between the high Ca^2+^ group and the low Ca^2+^ group. Additionally, the high Ca^2+^ group showed significantly less patients treated with ATG and a significantly different Karnofsky score. Therefore subsequent multivariate analysis included these variables.

### Incidence of Acute and Chronic GVHD

**FIGURE 4:**
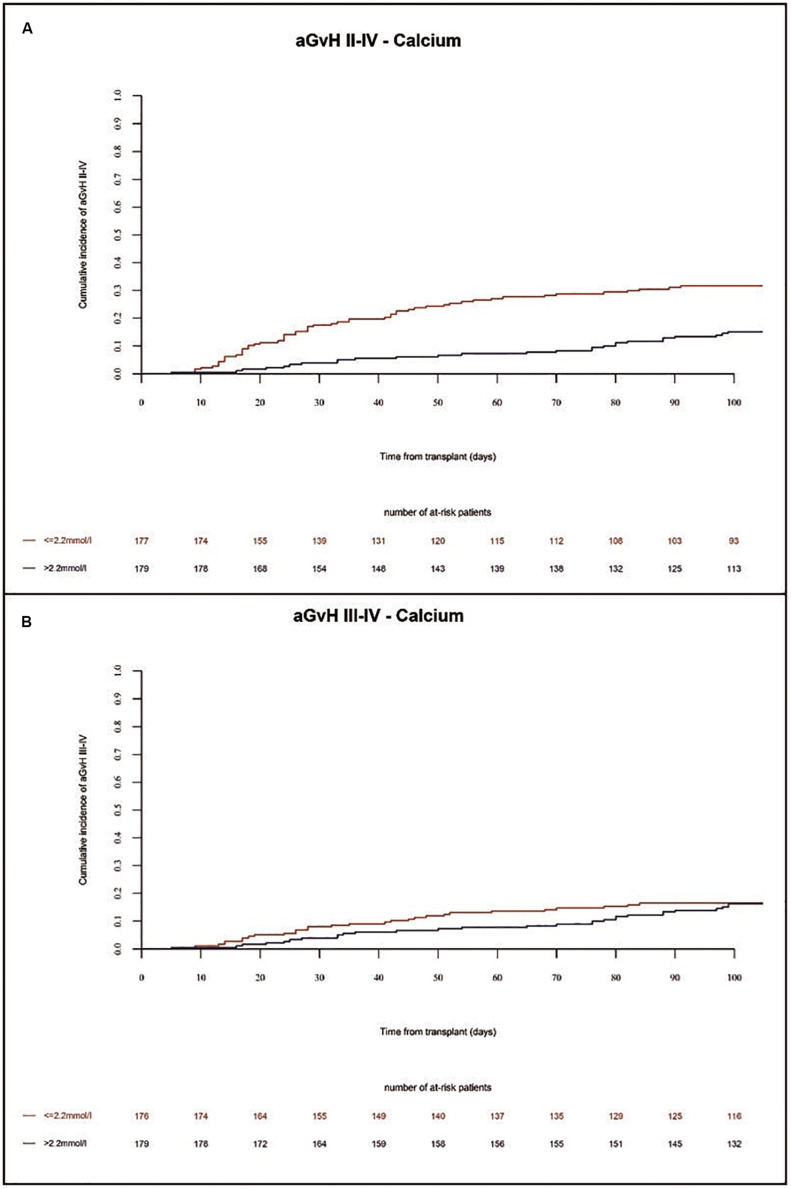
Incidence of acute GVHD until 100 days after alloSCT according to Ca^2+^ serum levels prior to alloSCT. Acute GVHD incidence of grades II-IV **(A)** and grades III-IV **(B)** was increased in patients with low Ca^2+^ levels (≤2.2 mmol/l, red line) as compared to patients with high Ca^2+^ levels (>2.2 mmol/l, blue line).

In the present study, the incidence of acute GVHD grades II-IV and grades III-IV in the whole population at 100 days were 25% and 11%, respectively. We found a higher incidence of acute GVHD grades II-IV in univariate (*p* = 0.0024) and multivariate (HR = 2.3 Cl = 1.45–3.85 *p* = 0.0006) analysis in patients with low Ca^2+^ serum levels before alloSCT as compared to patients with high Ca^2+^ serum levels (Figure 4A and Tables 1, 2). Accordingly, we found significant higher frequency of severe acute GVHD grades III-IV in univariate (*p* = 0.0012) and multivariate (HR = 3.3 CI = 1.59–7.14, *p* = 0.002) analysis in patients with low Ca^2+^ serum levels before alloSCT as compared to patients with high Ca^2+^ serum levels (Figure 4B and Tables 1, 2). Detailed numbers of aGVHD organ grades are available for 33% of patients with liver and skin aGVHD grades and 17% of patients with gut aGVHD grades (Supplementary Table 2).

**TABLE 1:** Univariate global comparison of acute GVHD shown at day + 100 after alloSCT.

**Group**	**Acute GVHDII–IV [95% CI]**	**Acute GVHD III–IV [95% CI]**
Serum Calcium ≤ 2.2 mmol/l	32% (25–39)	16% (11–22)
Serum Calcium > 2.2 mmol/l	18% (13–24)	6% (3–10)
*p*-value	*P* = 0.0024	*P* = 0.0012

**TABLE 2:** Multivariate global comparison.

**Calcium ≤ 2.2 mmol/l vs. >2.2 mmol/l**	**HR**	**95% CI**	***p*-value**
Overall survival	1,02	0,65 1,59	0.9443
Progression free survival	1,28	0,86 1,89	0.22
Relapse incidence	0,95	0,60 1,52	0.84
Non-relapse mortality	1,75	0,98 3,13	0.06
Chronic GVHD	1,52	0,98 2,38	0.06
Extensive chronic GVHD	2,0	1,04 3,85	0.04
Acute GVHD II-IV	2,33	1,45 3,85	0.0006
Acute GVHD III-IV	3,33	1,59 7,14	0.002

**FIGURE 5:**
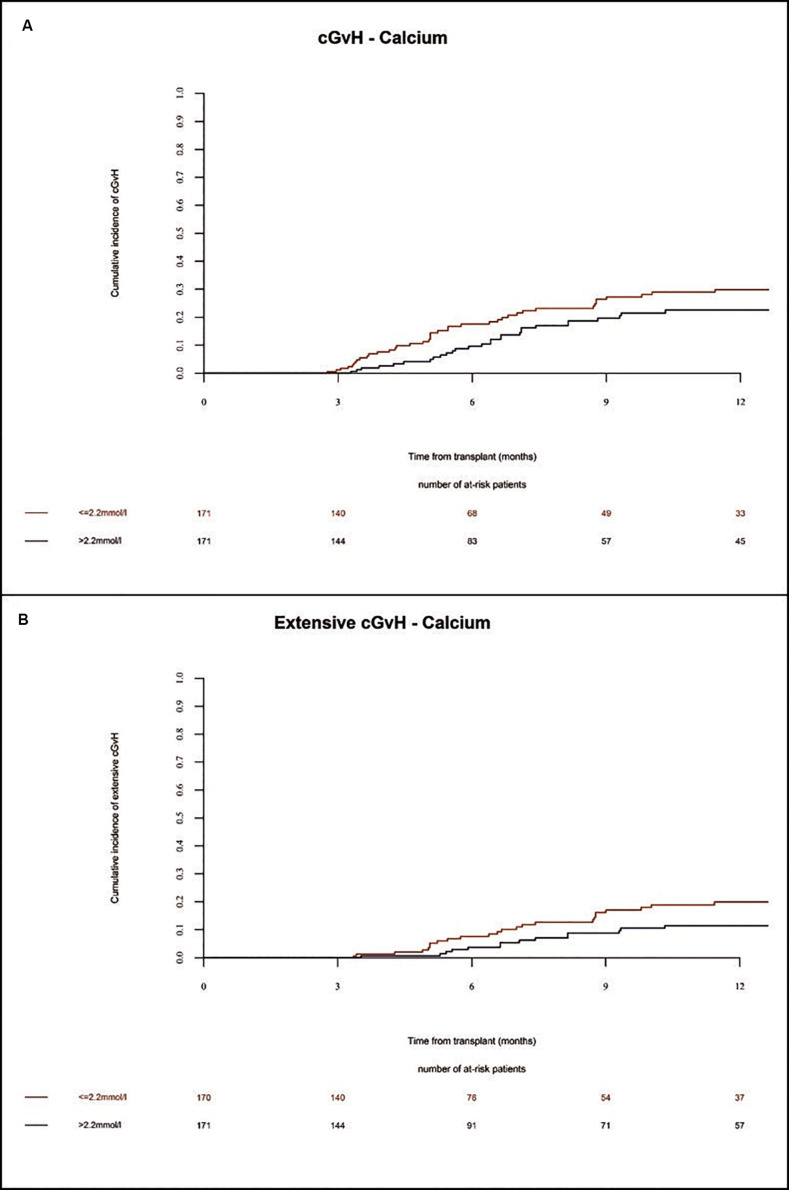
Incidence of chronic GVHD until 12 months after alloSCT according to Ca^2+^ serum levels prior to alloSCT. Incidence of chronic GVHD **(A)** and extensive chronic GVHD **(B)** was increased in patients with low Ca^2+^ levels (≤2.2 mmol/l, red line) as compared to patients with high Ca^2+^ levels (>2.2 mmol/l, blue line).

In the whole population, the incidence of chronic GVHD at 1 year and 2 years was 26% and 39%, respectively. The incidence of severe chronic GVHD at 1 year and 2 years was 16% and 21%, respectively. Extensive chronic GVHD was significantly increased in multivariate (HR = 2.0 Cl = 1.04–3.85 *p* = 0.04) analysis in patients with low Ca^2+^ serum levels before alloSCT as compared to patients with high Ca^2+^ serum levels (Figures 5A,B and Table 2). However, we observed a trend but no significant differences in overall incidence of chronic GVHD between the two cohorts. As expected, the chronic GVHD incidence was significantly lower in alloSCT recipients receiving anti-T-cell globulin as part of the conditioning regimen (ATG, HR = 0.25 CI = 0.13–0.5, *p* < 0.0001). NIH classification of chronic GVHD was available for 14% of patients shown in Supplementary Table 3.

### Survival Endpoints

**FIGURE 6:**
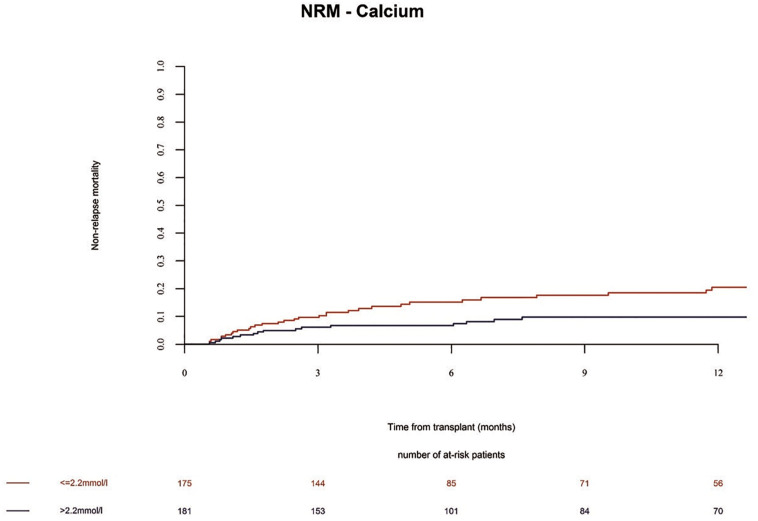
Non-relapse mortality (NRM) until 12 months after alloSCT according to Ca^2+^ serum levels prior to alloSCT. NRM was increased in patients with low Ca^2+^ levels (≤2.2 mmol/l, red line) as compared to patients with high Ca^2+^ levels (> 2.2 mmol/l, blue line).

The overall survival (OS) in the whole population at 1 year was 72%. We observed no significant differences in the OS and progression free survival (PFS) of alloSCT recipients with low Ca^2+^ serum levels before alloSCT as compared to patients with high Ca^2+^ serum levels (Table 2 and Supplementary Table 4, OS univariate *p* = 0.16; OS multivariate HR = 1.02 Cl = 0.65–1.59 *p* = 0.94 and PFS univariate *p* = 0.12; PFS multivariate HR = 1.28 Cl = 0.86–1.89 *p* = 0.22). However, non-relapse mortality (NRM) was significantly increased in alloSCT recipients with low Ca^2+^ levels prior to start of conditioning in univariate analysis (Figure 6 and Supplementary Table 4, *p* = 0.0247). The same trend (but not significant) was seen in NRM multivariate analysis (Table 2, HR = 1.75 Cl = 0.98–3.13 *p* = 0.06). The causes of death in patients without relapse were mainly due to infection. A descriptive analysis of the causes of death is given in Supplementary Table 5.

### Incidence of Relapse

No difference in the relapse incidence was detected between alloSCT recipients with low and high Ca^2+^ levels (Table 2 and Supplementary Table 4, univariate *p* = 0.8408; multivariate HR = 0.95, CI = 0.6–1.52, *p* = 0.84).

We conclude that low Ca^2+^ levels (below median of 2.2 mmol/l) prior to alloSCT are associated with increased incidence of acute GVHD as well as extensive chronic GVHD.

## Discussion

Ca^2+^ signaling is involved in various biologic processes relevant for patients after alloSCT, including inflammation, anti-infectious immunity, anti-tumor effects, kidney diseases and bone homeostasis. Although our current descriptive experimental and clinical results exhibit some limitations and lacks further mechanistic insights, it gives first insights in a possible association between Ca^2+^ and the occurrence of GVHD, deserving further investigations.

In animal experiments, GPRC6a^–/–^ alloSCT recipients had increased GVHD morbidity and mortality. GPRC6a, a G protein coupled receptor, was previously found to be activated by increased extracellular Ca^2+^ levels leading to an increase in the intracellular Ca^2+^ concentration, triggering the activation of i.a. the NLRP3 inflammasome and caspase-1, mediated through the phosphatidyl inositol/Ca^2+^ pathway. Activation of the NLRP3 inflammasome via DAMPs has already been shown to be connected to GVHD severity (7, 19). In preclinical models, inhibition of the NLRP3 inflammasome lead to improved GVHD morbidity and delayed GVHD-induced mortality (5–7). Interestingly, when using NLRP3 deficient mice, this effect was only prominent when deficiency was present in alloSCT recipients, not in donors (6). In patients, recipient single nucleotide polymorphisms of NLRP3 were found connected to the incidence of acute GVHD grades 2–4 (20). While the role of NLRP3 was studied in GVHD, potential upstream immune regulatory functions of GPRC6a are unknown. Results in GPRC6a^–/–^ mice showing reduced inflammatory response and reduced inflammasome activation in a mouse model of carrageenan-induced footpad swelling (4), suggested that GPRC6a deficiency may also lead to reduced GVHD-associated inflammation as seen in GVHD studies analyzing the NLRP3 inflammasome. However, our data contradicts these findings, as GPRC6a deficiency in alloSCT recipients was associated to increased acute GVHD. As GPRC6a is located upstream from the NLRP3 inflammasome, influence on other pathways is likely, potentially leading to differing results. In addition, GPRC6a is widely expressed throughout the body and can recognize multiple ligands next to Ca^2+^, e.g., L-α-amino acids as L-arginine or L-lysin, zinc, testosterone or osteocalcin acting as positive or negative allosteric modulators (9, 21). The wide tissue expression and multiple activators of GPRC6a, let assume a wide range of functions and the entire scope of GPRC6a functions is not yet known. In line, experimental data from GPRC6a^–/–^ mice show a diversity in physiological and pathophysiological functions, mainly involving a role in inflammation, endocrine functions and metabolism. However, functional phenotyping of these mice is enclosed by disparities, e.g., in glucose and fat or bone metabolism (9, 22). Whereas one group found glucose intolerance and insulin resistance as well as osteopenia and impaired osteoblast mediated bone formation in GPRC6a^–/–^ mice (22), other groups could not detect such phenotypes (23, 24). This underlines the possibility that besides the Ca^2+^ mediated GPRC6a signaling, other signaling pathways contribute to the observed phenotype in our GVHD model.

Of note, despite its regulatory functions in inflammation, GPRC6a was shown to mediate the non-genomic effects of testosterone and other steroids *in vitro* and in mice (25). This raises the possibility that steroid therapy used for GVHD prophylaxis may be influenced by GPRC6a expression.

GPRC6a can be directly activated by Ca^2+^ in concentrations above 5 mM (9); and Ca^2+^ was shown to be involved in various immune functions, including T-cell effector and regulatory functions (1) as well as the allo-activation of T-lymphocytes by antigen presenting cells (2, 3). In GPRC6a^–/–^ mice, we also found increased antigen presentation potential demonstrated by higher proliferation rates of allogeneic T-lymphocytes in GPRC6a^–/–^ compared to wildtype alloSCT recipients, leading to increased GVHD severity. Phenotypic differences of GPR6CA^–/–^ dendritic cells were detected concerning reduced CD80 expression; however, further changes in costimulatory molecules are likely and need further investigation. The role of Ca^2+^ signaling during GVHD is mostly unknown, although first results in preclinical GVHD models support our data that Ca^2+^ may play a role in mediating GVHD pathophysiology (3, 11). However, Ca^2+^ signaling appears to remain complex, multi-targetable and divergent and deserves further investigation. E.g., the deletion of the plasma membrane proteins ORAI1 + 2 forming release-activated Ca^2+^ channels leads to the abolishment of store-operated Ca^2+^ entry and a decrease of intracellular Ca^2+^, and protects mice from allo-immunity in models of colitis and GVHD (3). Contrasting and more coherent to our data, the inhibition of inositol 1,4,5-trisphosphate 3-kinase B (Itpkb), a negative regulator of Ca^2+^ influx, leads to an increase of intracellular Ca^2+^ in donor T cells, protecting mice from acute and chronic GVHD while maintaining a graft-versus-tumor-effect (11). Interestingly, high calcium containing solutions are effective to reduce mucositis after alloSCT raising the possibility that Ca^2+^ signaling is involved in tissue repair or tissue protection (26).

Our performed prospective study suggests an association of Ca^2+^ with the occurrence of GVHD as low Ca^2+^ serum levels (below median of 2.2 mmol/l) prior to alloSCT were found to be a risk factor for the incidence of acute GVHD and for extensive chronic GVHD. However, a limitation of this clinical study is the lack of Ca^2+^ serum levels after alloSCT, e.g., during occurrence of GVHD; leading to an incomplete understanding of the effects of Ca^2+^ on post alloSCT immunity. The aim of our prospective trial was to identify biomarkers that can be measured before alloSCT. Therefore, no biomarker measurement at time of GVHD onset was planned in the trial design. Importantly, further investigations addressing the longitudinal progress of Ca^2+^ levels post alloSCT will further shed light on the association of Ca^2+^ and GVHD pathophysiology. In this study, we assessed total Ca^2+^ in serum corrected to albumin levels, as approximate 45% of circulating Ca^2+^ is bound to proteins, mainly albumin, which displays besides its high-capacity carrier role also several physiological functions. Despite this correction, subsequent analyses may address if free ionized Ca^2+^ and/or Ca^2+^ bound to proteins or anions show an impact on GVHD incidence. Of note, we found in our population a strong correlation between calcium and albumin values (*r* = 0.56, *p* < 0.0001). Recently, hypoalbuminemia was shown to predict worse NRM and inferior OS (27) as well as severity of aGVHD (28) in alloSCT recipients.

Our patient population was restricted to alloSCT from HLA-identical sibling donors, allowing to draw conclusions from a homogeneous population with similar GVHD prophylaxis strategies and similar incidences of GVHD and other complications. However, this includes the limitation that we are therefore unable to draw definite conclusions from these results regarding the association of Ca^2+^ serum levels with outcome in matched unrelated donor alloSCT or in haploidentical alloSCT, which are increasingly used. The herein presented data can stimulate further research to perform analyses of Ca^2+^ serum levels in other multiple cohorts allowing to widen conclusions to other donor types.

## Conclusion

This study gives first insights in a possible association of Ca^2+^ with the incidence and severity of GVHD. Future perspectives of our results will include the clinical implementation of risk-adapted GVHD prophylaxis strategies taking into account calcium levels before alloSCT. Future studies can address the question how the high risk of GVHD in the subpopulation identified in this study can be reduced. A possible trial design would be to test intensified GVHD prophylaxis regimens in a randomized trial, based on calcium levels. This intensified regimen could be the addition of a third immunosuppressive drug in addition to the standard regimens consisting of a calcineurin inhibitor and an antimetabolite. Furthermore, future research areas of interest include: 1) to further analyze the involved mechanistic and molecular pathways of Ca^2+^-dependent allo-activation, and 2) to test if Ca^2+^ signaling can serve as a therapeutic target during GVHD or other inflammatory diseases.

## Data Availability Statement

The raw data supporting the conclusions of this article will be made available by the authors, without undue reservation.

## Ethics Statement

The studies involving human participants were reviewed and approved by IRB and/or Ethics Committee in all participating centers. The patients/participants provided their written informed consent to participate in this study. The animal study was reviewed and approved by State Office of Health and Social Affairs (LAGeSo), Berlin.

## Author Contributions

KR, SC, and OP designed the animal study and wrote the manuscript. SC, MK, SM, and JM performed experimental GVHD experiments. CS and YS performed and analyzed qPCR experiments. KR and SC analyzed experimental data. OP, ZP, CK, HS, RD, and GB designed and performed the EBMT study. CP and SW analyzed human data and performed statistical analyses. All authors contributed to the article and approved the submitted version.

## Conflict of Interest

The authors declare that the research was conducted in the absence of any commercial or financial relationships that could be construed as a potential conflict of interest.

## Footnotes

1 https://www.R-project.org/

## References

[1] Feske S. Calcium signalling in lymphocyte activation and disease. *Nat Rev Immunol.* (2007) 7:690–702. 10.1038/nri2152 17703229

[2] Manes TD Wang V Pober JS. Divergent TCR-initiated calcium signals govern recruitment versus activation of human alloreactive effector memory T cells by endothelial cells. *J Immunol.* (2018) 201:3167–74. 10.4049/jimmunol.1800223 30341183 PMC6246797

[3] Vaeth M Yang J Yamashita M Zee I Eckstein M Knosp C ORAI2 modulates store-operated calcium entry and T cell-mediated immunity. *Nat Commun.* (2017) 8:14714. 10.1038/ncomms14714 28294127 PMC5355949

[4] Rossol M Pierer M Raulien N Quandt D Meusch U Rothe K Extracellular Ca2+ is a danger signal activating the NLRP3 inflammasome through G protein-coupled calcium sensing receptors. *Nat Commun.* (2012) 3:1329. 10.1038/ncomms2339 23271661 PMC3535422

[5] Chen S Smith BA Iype J Prestipino A Pfeifer D Grundmann S MicroRNA-155-deficient dendritic cells cause less severe GVHD through reduced migration and defective inflammasome activation. *Blood.* (2015) 126:103–12. 10.1182/blood-2014-12-617258 25972159

[6] Jankovic D Ganesan J Bscheider M Stickel N Weber FC Guarda G The Nlrp3 inflammasome regulates acute graft-versus-host disease. *J Exp Med.* (2013) 210:1899–910. 10.1084/jem.20130084 23980097 PMC3782050

[7] Koehn BH Zeiser R Blazar BR. Inflammasome effects in GvHD. *Oncotarget.* (2015) 6:38444–5. 10.18632/oncotarget.6307 26564963 PMC4770712

[8] Piper C Drobyski WR. Inflammatory cytokine networks in gastrointestinal tract graft vs host disease. *Front Immunol.* (2019) 10:163. 10.3389/fimmu.2019.00163 30853956 PMC6395399

[9] Clemmensen C Smajilovic S Wellendorph P Brauner-Osborne H. The GPCR, class C, group 6, subtype A (GPRC6A) receptor: from cloning to physiological function. *Br J Pharmacol.* (2014) 171:1129–41. 10.1111/bph.12365 24032653 PMC3952793

[10] Dehghan A Dupuis J Barbalic M Bis JC Eiriksdottir G Lu C Meta-analysis of genome-wide association studies in >80 000 subjects identifies multiple loci for C-reactive protein levels. *Circulation.* (2011) 123:731–8. 10.1161/CIRCULATIONAHA.110.948570 21300955 PMC3147232

[11] Thangavelu G Du J Paz KG Loschi M Zaiken MC Flynn R Inhibition of inositol kinase B controls acute and chronic graft-versus-host disease. *Blood.* (2020) 135:28–40. 10.1182/blood.2019000032 31697815 PMC6940197

[12] Mengwasser J Babes L Cordes S Mertlitz S Riesner K Shi Y Cathepsin E deficiency ameliorates graft-versus-host disease and modifies dendritic cell motility. *Front Immunol.* (2017) 8:203. 10.3389/fimmu.2017.00203 28298913 PMC5331043

[13] Mertlitz S Shi Y Kalupa M Grotzinger C Mengwasser J Riesner K Lymphangiogenesis is a feature of acute GVHD, and VEGFR-3 inhibition protects against experimental GVHD. *Blood.* (2017) 129:1865–75. 10.1182/blood-2016-08-734210 28096093

[14] Riesner K Shi Y Jacobi A Krater M Kalupa M McGearey A Initiation of acute graft-versus-host disease by angiogenesis. *Blood.* (2017) 1 :2021–32. 10.1182/blood-2016-08-736314 28096092

[15] Nogai A Shi Y Perez-Hernandez D Cordes S Mengwasser J Mertlitz S Organ siderosis and hemophagocytosis during acute graft-versus-host disease. *Haematologica.* (2016) 101:e344–6. 10.3324/haematol.2016.144519 27198715 PMC4967586

[16] Riesner K Kalupa M Shi Y Elezkurtaj S Penack OA. preclinical acute GVHD mouse model based on chemotherapy conditioning and MHC-matched transplantation. *Bone Marrow Transplant.* (2016) 51:410–7. 10.1038/bmt.2015.279 26595081

[17] Przepiorka D Weisdorf D Martin P Klingemann HG Beatty P Hows J 1994 consensus conference on acute GVHD grading. *Bone Marrow Transplant.* (1995) 15:825–8. 7581076

[18] Lee SJ Vogelsang G Flowers ME. Chronic graft-versus-host disease. *Biol Blood Marrow Transplant.* (2003) 9:215–33. 10.1053/bbmt.2003.50026 12720215

[19] Wilhelm K Ganesan J Muller T Durr C Grimm M Beilhack A Graft-versus-host disease is enhanced by extracellular ATP activating P2X7R. *Nat Med.* (2010) 16:1434–8. 10.1038/nm.2242 21102458

[20] Takahashi H Okayama N Yamaguchi N Miyahara Y Morishima Y Suehiro Y Associations of interactions between NLRP3 SNPs and HLA mismatch with acute and extensive chronic graft-versus-host diseases. *Sci Rep.* (2017) 7:13097. 10.1038/s41598-017-13506-w 29026154 PMC5638959

[21] Pi M Nishimoto SK Quarles LD. GPRC6A: jack of all metabolism (or master of none). *Mol Metab.* (2017) 6:185–93. 10.1016/j.molmet.2016.12.006 28180060 PMC5279936

[22] Pi M Chen L Huang MZ Zhu W Ringhofer B Luo J GPRC6A null mice exhibit osteopenia, feminization and metabolic syndrome. *PLoS One.* (2008) 3:e3858. 10.1371/journal.pone.0003858 19050760 PMC2585477

[23] Smajilovic S Clemmensen C Johansen LD Wellendorph P Holst JJ Thams PG The L-alpha-amino acid receptor GPRC6A is expressed in the islets of Langerhans but is not involved in L-arginine-induced insulin release. *Amino Acids.* (2013) 44:383–90. 10.1007/s00726-012-1341-8 22714012

[24] Wellendorph P Johansen LD Jensen AA Casanova E Gassmann M Deprez P No evidence for a bone phenotype in GPRC6A knockout mice under normal physiological conditions. *J Mol Endocrinol.* (2009) 42:215–23. 10.1677/JME-08-0149 19103720

[25] Pi M Parrill AL Quarles LD. GPRC6A mediates the non-genomic effects of steroids. *J Biol Chem.* (2010) 285:39953–64. 10.1074/jbc.M110.158063 20947496 PMC3000977

[26] Bhatt N Naithani R Gupta SK. Supersaturated calcium phosphate rinse in prevention and treatment of mucositis in patients undergoing hematopoietic stem cell transplant. *Exp Clin Transplant.* (2017) 15:567–70. 10.6002/ect.2016.0180 28229803

[27] Vaughn JE Storer BE Armand P Raimondi R Gibson C Rambaldi A Design and validation of an augmented hematopoietic cell transplantation-comorbidity index comprising pretransplant ferritin, albumin, and platelet count for prediction of outcomes after allogeneic transplantation. *Biol Blood Marr Transplant.* (2015) 21:1418–24. 10.1016/j.bbmt.2015.04.002 25862589 PMC4506728

[28] Kharfan-Dabaja MA Sheets K Kumar A Murthy HS Nishihori T Tsalatsanis A Hypoalbuminaemia segregates different prognostic subgroups within the refined standard risk acute graft-versus-host disease score. *Br J Haematol.* (2018) 180:854–62. 10.1111/bjh.15105 29345306

