# Supplementary material for: Reduced Calcium Signaling Is Associated With Severe Graft-Versus-Host Disease: Results From Preclinical Models and From a Prospective EBMT Study
Source: Front Immunol. 2020 Aug 11;11:1983. doi: 10.3389/fimmu.2020.01983 (PMC7431962; doi:10.3389/fimmu.2020.01983)
Supplement: Supplementary file 1 [file Table_1.DOCX]

Supplementary Material

**Supplementary Tables**

**Supplementary Table 1. Patient characteristics.**

|  | Serum Calcium <=2.2mmol/l  (n=177) | Serum Calcium >2.2mmol/l  (n=183) | p-value |
| --- | --- | --- | --- |
| Year of transplant median (range) [IQR] | 2015 (2014-2018)  [ 2015-2016 ] | 2015 (2014-2018)  [ 2015-2016 ] | 0.915 |
| Patient age (years) median(range) [IQR] | 54.5 (19.7-71.3)  [ 42.9-62.5 ] | 51.7 (17.1-71)  [ 42.1-59.8 ] | 0.0957 |
| Time from diagnosis to transplant (months) median(range)[IQR] | 4 (1-62)  [ 3-7.5 ] | 5 (1-71)  [ 3.2-6 ] | 0.4493 |
| Number of CD34+ cells infused (e+06) median (range)[IQR] | 5.79 (0.63-11)  [ 4.47-6.98 ] | 5.9 (0.9-10.7)  [ 4.67-7.3 ] | 0.3769 |
| **Sex mismatch** |  |  | 0.2232 |
| Female to male | 45 ( 26.32% ) | 37 ( 20.79% ) |  |
| Other combination | 126 ( 73.68% ) | 141 ( 79.21% ) |  |
| Missing | 6 | 5 |  |
| **Diagnosis** |  |  | 0.1839 |
| Acute leukaemia | 112 ( 63.28% ) | 131 ( 71.58% ) |  |
| Lymphoma | 23 ( 12.99% ) | 15 ( 8.2% ) |  |
| Myelodysplastic/Myeloproliferative | 42 ( 23.73% ) | 37 ( 20.22% ) |  |
| **Disease status** |  |  | < 0.0001 |
| CR | 95 ( 54.91% ) | 143 ( 81.71% ) |  |
| Not in CR | 78 ( 45.09% ) | 32 ( 18.29% ) |  |
| missing | 4 | 8 |  |
| **DRI** |  |  | 0.06 |
| low | 5 ( 3.01% ) | 9 ( 5.23% ) |  |
| Intermediate | 95 ( 57.23% ) | 116 ( 67.44% ) |  |
| High | 56 ( 33.73% ) | 43 ( 25% ) |  |
| very high | 10 ( 6.02% ) | 4 ( 2.33% ) |  |
| missing | 11 | 11 |  |
| **ATG** |  |  | 0.0002 |
| no | 81 ( 45.76% ) | 119 ( 65.03% ) |  |
| yes | 96 ( 54.24% ) | 64 ( 34.97% ) |  |
| **Conditioning Intensity** |  |  | 0.0296 |
| MAC/Chemo | 32 ( 18.39% ) | 43 ( 23.89% ) |  |
| MAC/TBI | 23 ( 13.22% ) | 38 ( 21.11% ) |  |
| RIC | 119 ( 68.39% ) | 99 ( 55% ) |  |
| missing | 3 | 3 |  |
| **GvHD prophylaxis** |  |  | < 0.0001 |
| Calcineurin inhibitor + MMF | 102 ( 60% ) | 67 ( 38.07% ) |  |
| Calcineurin inhibitor + MTX | 44 ( 25.88% ) | 82 ( 46.59% ) |  |
| Calcineurin inhibitor monotherapy | 24 ( 14.12% ) | 27 ( 15.34% ) |  |
| missing | 7 | 7 |  |
| **Donor/patient CMV** |  |  | 0.388 |
| -/- | 37 ( 22.02% ) | 32 ( 18.29% ) |  |
| Other | 131 ( 77.98% ) | 143 ( 81.71% ) |  |
| missing | 9 | 8 |  |
| **Karnofsky score** |  |  | 0.0109 |
| 10-80 | 40 ( 23.53% ) | 23 ( 12.99% ) |  |
| 90-100 | 130 ( 76.47% ) | 154 ( 87.01% ) |  |
| missing | 7 | 6 |  |
| HT-CI = 0 | 52 ( 42.28% ) | 61 ( 44.85% ) | 0.7533 |
| HT-CI = 1 or 2 | 23 ( 18.7% ) | 28 ( 20.59% ) |  |
| HT-CI >=3 | 48 ( 39.02% ) | 47 ( 34.56% ) |  |
| missing | 54 | 47 |  |

**Supplementary Table 2. aGVHD by organs. Data from MED-A forms.**

| Variable | Level | Serum Calcium <=2.2mmol/l  (n=177) | Serum Calcium >2.2mmol/l  (n=183) | Overall  (n=360) |
| --- | --- | --- | --- | --- |
| Skin aGVHD grade | **0** | **15 (20.8%)** | **8 (17.4%)** | **23 (19.5%)** |
|  | **1** | **21 (29.2%)** | **17 (37%)** | **38 (32.3%)** |
|  | **2** | **19 (26.4%)** | **15 (32.6%)** | **34 (28.8%)** |
|  | **3** | **15 (20.8%)** | **6 (13 %)** | **21 (17.8 %)** |
|  | **4** | **2 (2.8%)** | **0 (0%)** | **2 (1.7%)** |
|  | **missing** | **105** | **137** | **242 (67,2%)** |
| Liver aGVHD grade | **0** | **59 (80.8%)** | **43 (93,5%)** | **102 (85.7%)** |
|  | **1** | **6 (8.2%)** | **1 (2,2%)** | **7 (5.9%)** |
|  | **2** | **4 (5.5%)** | **1 (2.2%)** | **5 (4.2%)** |
|  | **3** | **1 (1.4%)** | **0 (0%)** | **1 (0.8%)** |
|  | **4** | **3 (4.1%)** | **1 (2.2%)** | **4 (3.4%)** |
|  | **missing** | **104** | **137** | **241 (66.9%)** |
| Gut aGVHD grade | **0** | **9 (27.3%)** | **14 (46.7%)** | **23 (36.5%)** |
|  | **1** | **8 (24.2%)** | **9 (30%)** | **17 (27%)** |
|  | **2** | **4 (12.1%)** | **4 (13.3%)** | **8 (12.7%)** |
|  | **3** | **6 (18.2%)** | **2 (6.7%)** | **8 (12.7%)** |
|  | **4** | **6 (18.2%)** | **1 (3.3%)** | **7 (11.1%)** |
|  | **missing** | **144** | **153** | **297 (82.5%)** |

**Supplementary Table 3. NIH classification of cGVHD. Data from MED-A forms.**

| Variable | Level | Serum Calcium <=2.2mmol/l  (n=177) | Serum Calcium >2.2mmol/l  (n=183) | Overall  (n=360) |
| --- | --- | --- | --- | --- |
| NIH Maximum cGVHD grade | **mild** | **10 (32.3%)** | **9 (45%)** | **19 (37.3%)** |
|  | **moderate** | **16 (51.6%)** | **11 (55%)** | **27 (52.9%)** |
|  | **severe** | **5 (16.1%)** | **0 (0%)** | **5 (9.8%)** |
|  | **missing** | **146** | **163** | **309 (85.8 %)** |

**Supplementary Table 4. Univariate global comparison of OS, NRM, relapse incidence (RI) as well as chronic GVHD incidence and severity at one year after alloSCT.**

| **Group** | **OS**  **[95% CI]** | **PFS**  **[95% CI]** | **RI**  **[95% CI]** | **NRM**  **[95% CI]** | **cGVHD**  **[95% CI]** | **Extensive cGVHD [95% CI]** |
| --- | --- | --- | --- | --- | --- | --- |
| **Serum Calcium <=2.2mmol/l** | **67%**  **(59-75)** | **58%**  **(50-67)** | **22%**  **(15-29)** | **20%**  **(14-27)** | **30%**  **(22-38)** | **20%**  **(13-28)** |
| **Serum Calcium >2.2mmol/l** | **76%**  **(70-84)** | **66%**  **(59-74)** | **24%**  **(17-31)** | **10%**  **(6-15)** | **22%**  **(16-30)** | **11%**  **(7-18)** |
| **p-value** | **p = 0.16** | **p = 0.123** | **p = 0.8408** | **p = 0.0247** | **p = 0.3425** | **p = 0.1499** |

**Supplementary Table 5. Causes of death in both cohorts.**

|  | Serum Calcium <=2.2mmol/l  (n=177) | Serum Calcium >2.2mmol/l  (n=186) |
| --- | --- | --- |
| Alive at last follow up | **119 (67.2% )** | **134 (74.4% )** |
| Dead | **58 (33.8%)** | **52 (25.6%)** |
| Dead due to relapse | **23 (13.0%)** | **28 (15.6%)** |
| Dead without relapse | **35 (20.8%)** | **24 (10.0%)** |
| Dead due to infection | **19 (13%)** | **10 (5.4%)** |
| Dead due to GVHD | **9 (3.2%)** | **2 (1.1%)** |
| Dead due to infection and GVHD | **7 (2.5%)** | **2 (1.1%)** |
| Dead due to other causes or unknown | **23 (8.3%)** | **35 (18.8%)** |
